# Editorial: Exploration of decision neuroscience research in the digital era

**DOI:** 10.3389/fpsyg.2025.1682163

**Published:** 2025-10-30

**Authors:** Wei Shan, Jing Luan, Richard Evans

**Affiliations:** ^1^School of Economics and Management, Beihang University, Beijing, China; ^2^School of Economics and Management, Beijing Jiaotong University, Beijing, China; ^3^College of Digital Transformation, Faculty of Computer Science, Dalhousie University, Halifax, NS, Canada

**Keywords:** digital era, decision neuroscience, decision-making scenarios, behavior, multimodal

## 1 Introduction

The digital era has revolutionized the way we study human decision-making. Advances in neuroimaging, computational modeling, and machine learning have provided insights into these complex processes. This Research Topic, “*Exploration of Decision Neuroscience Research in the Digital Era*”, brings together cutting-edge studies that leverage modern technologies—such as eye-tracking, neuroimaging, digital dynamic assessment, and generative narrative survey—to examine the neural and behavioral underpinnings of decision-making.

## 2 Contributions to the Research Topic

The articles featured in this Research Topic illustrate the breadth of current approaches: Huang et al. introduced a maze-based digital assessment paradigm to detect early cognitive decline in Parkinson's disease; Wong et al. applied generative narrative surveys to capture real-world decision-making in varied social contexts; Zhou et al. employed eye-tracking to study intertemporal loss decisions; and Horr et al. demonstrated how machine learning applied to EEG signals can accurately predict online purchasing behavior.

